# Diagnostic Accuracy of a Thick Blood Smear Compared to qPCR for Malaria Associated with Pregnancy in Colombia

**DOI:** 10.3390/tropicalmed8020119

**Published:** 2023-02-14

**Authors:** Jaiberth Antonio Cardona-Arias, Luis Felipe Higuita Gutiérrez, Jaime Carmona-Fonseca

**Affiliations:** 1 Escuela de Microbiología, Universidad de Antioquia, Medellín 050010, Colombia; 2 Facultad de Medicina, Universidad Cooperativa de Colombia, Medellín 050010, Colombia; 3 Grupo Salud y Comunidad César Uribe Piedrahíta, Facultad de Medicina, Universidad de Antioquia, Medellín 050010, Colombia

**Keywords:** gestation, placenta, congenital, malaria, thick blood smear, qPCR, diagnostic accuracy

## Abstract

This study aimed to evaluate the accuracy of the thick blood smear (TBS) versus quantitative polymerase chain reaction (qPCR) for the diagnosis of malaria associated with pregnancy (MAP) caused by *P. falciparum* or *P. vivax* in Colombia in its gestational malaria (GM), placental malaria (PM), and congenital malaria (CM) forms as well as to compare its accuracy in different subgroups of pregnant women according to the presence of fever, anemia and a history of malaria. This was a diagnostic evaluation of 829 pregnant women, 579 placentas, 381 umbilical cord samples, and 221 neonatal peripheral blood samples. Accuracy was evaluated based on the parameters of sensitivity, specificity, predictive values, likelihood ratios, and validity index, with their 95% confidence intervals. The frequency of GM was 36% (*n* = 297/829), PM 27% (*n* = 159/579), and CM 16.5% (*n* = 63/381) in umbilical cord samples and 2% (*n* = 5/221) in neonatal peripheral blood samples. For GM, the sensitivity was 55%, with higher rates in those infected with *P. vivax* (68%), with a history of malaria (69%), and with fever (96%). These three subgroups presented the best results in terms of the negative likelihood ratio and validity index. For PM, sensitivity was 8%; in subgroup analyses in terms of species, symptomatology (anemia and fever), and history of malaria, it was 1–18%, and the negative likelihood ratio was >0.80 in all subgroups. No false positives were recorded in any of the subgroups. The TBS did not detect any cases of CM. This study found the TBS yielded satisfactory results in terms of diagnosing GM for *P. vivax,* pregnant women with previous malaria and febrile. It also showed that the TBS is not useful for diagnosing PM and CM. It is necessary to conduct surveillance of MAP with molecular methods in in groups where TBS is deficient (asymptomatic GM, *P. falciparum*, and pregnant women without history of malaria) to optimize the timely treatment of PM and CM, avoid the deleterious effects of MAP and achieve the malaria elimination goals in Colombia.

## 1. Introduction

The World Health Organization (WHO) reported 247 million new cases of malaria in 2021 and 619,000 deaths [1]. Although this report does not include malaria associated with pregnancy (MAP), other publications estimate a high number of pregnant women exposed [2,3]. In Colombia, the population of pregnant women exposed to malaria is estimated to be 59,962 in 2022 [4].

MAP includes gestational malaria (GM) or *Plasmodium* spp. infection in maternal peripheral blood, placental malaria (PM) or the presence of the parasite or hemozoin in placental tissue, and congenital malaria (CM) in which the parasite is found in the newborn’s umbilical cord blood or peripheral blood discarding vector bite [5,6,7]. In Colombia, the coexistence of GM and PM is common, but not with CM [8].

According to the Centers for Disease Control and Prevention from United States of America, *Plasmodium falciparum* MAP in areas with high transmission produces an immune profile that prevents severe cases and generates high frequency of asymptomatic malaria, but it also causes *P. falciparum* cytoadhering to the placenta, thereby increasing the risk of PM and CM [9]. Women do not develop immunity to malaria in areas with low transmission, thereby increasing their risk of *Plasmodium* spp. infection and its clinical outcomes [2,9]. Less is known about *P. vivax* MAP but its risk of PM and CM is high. In Colombia, both species cause PM with similar lesions, histological changes and physiological mediators [10].

WHO experts on MAP indicates that pregnancy reduces immunity, with the following consequences: increased susceptibility to infection, risk of severe disease, likelihood of severe anemia and maternal death, risk of miscarriage, stillbirth, preterm delivery, low birth weight, and infant death [3].

WHO recommendations for MAP prevention are mainly focused on Africa through intermittent preventive treatment with sulfadoxyne-pyrimethamine. In other regions, MAP control is based on case management and the passive detection [3]. Therefore, WHO guidelines only recommend parasitological diagnosis for symptomatic individuals using light microscopy or rapid diagnostic tests in order to optimize the economic use of diagnostic resources [1,11].

In Colombia, there are few clinical–epidemiological studies on MAP [12]. A meta-analysis reported a prevalence of 16.7% (95% confidence interval [CI], 9.0–28.8) for GM, 11.0% (95% CI, 4.1–26.3) PM, and 16.2% (95% CI, 8.2–29.5) CM [13]. Another study found 35.8% GM, associated with maternal anemia, CM, and lower birth weight [14]. A study reported PM 27.7%, associated with CM, lower neonatal weight, and maternal anemia [15]. In 567 newborns, CM was 12.2%, associated with a lower birth weight [16].

Despite the high magnitude and serious consequences of MAP, Colombia lacks specific control strategies and only has a clinical practice guideline (CPG) for the diagnosis and treatment of malaria, which highlights the importance of early diagnosis for the control, elimination and preventing complications. It also explains the diagnosis is based on thick blood smears (TBS) and rapid diagnostic tests for patients who are symptomatic to avoid health costs [17].

Despite the indications of Colombian CPG, the Colombian National Institute of Health (NIH) recommends that pregnant women in endemic areas undergo TBS testing during their antenatal check-ups every trimester, even if they are asymptomatic. NIH emphasizes the importance of case finding based on both clinical (symptomatology) and epidemiological (based on exposure in an endemic area or contact with a case) criteria [18]. TBS is the reference method for malaria diagnosis in Colombia. It is a simple, low-cost test that allows species identification, parasite stage differentiation, parasitemia level determination, and treatment response monitoring; but it requires trained personnel and has limitations in the diagnosis of low parasitemia and mixed malaria. Its detection limit is 10–30 parasites/µL of blood. Another factor affecting the diagnostic performance of TBS is the pyrogenic threshold (minimal parasitemia necessary to produce fever), which is usually lower in patients lacking prior immunity [19].

The focus of malaria control on microscopic diagnosis is critical for MAP because there is a high frequency of asymptomatic and submicroscopic cases (negative by the TBS and positive by polymerase chain reaction [PCR]) in GM, PM, and CM [13]; they are not receiving treatment, thus increasing the risk of clinical outcomes of MAP and making endemic control and the prevention of *Plasmodium* spp. transmission impossible [20,21,22,23,24,25,26].

In addition, there are few studies on the diagnostic accuracy of the TBS for MAP. Some studies on asymptomatic pregnant women found low and poor sensitivity (32.3% 95% CI, 20.9–45.3) [27], in others was moderate (63.7% 95% CI, 56.1–70.9) [28]; and there are reports of sensitivity high in febrile pregnant women (94.4%, 95% CI, 72.7–99.9) [29]. In other contexts, satisfactory accuracy parameters have been reported for *P. falciparum* PM even in low (less than 1000 parasites/µL) and moderate (1000–10,000 parasites/µL) parasitemia, with sensitivity ranging between 76% and 78% [30].

Despite the TBS’s low sensitivity for MAP, it remains as the gold standard in Colombia because of the factual impossibility of implementing molecular diagnosis in rural areas (where there are highest number of cases of malaria and MAP in Colombia) [31]. According to a recent review, although TBS has been the standard for diagnosis globally, its sensitivity is low and is dependent on variables such as the microscopist’s experience, the quality of the slides, and the reagents. In addition, its low accuracy in low endemic areas, in patients with low parasitemia, and in patients with submicroscopic and asymptomatic infections, prevents the achievement of elimination goals [32]. Therefore, TBS is useful for the programmatic phase of malaria control (focusing on reducing incidence, prevalence, morbidity, and mortality) but not for the following three phases: pre-elimination (rates below 5%, high coverage of a good quality control program in the clinical laboratories and medical services, and enhanced case reporting and surveillance), elimination (absence of cases), and prevention of reintroduction [32].

Additionally, the central strategy has been active case detection in countries that have advanced to the elimination phases, in which high-risk individuals in an area or household members, neighbors, and other contacts of passively detected cases (hotspots and hotpops) are screened and treated in conjunction with vector control actions [33,34,35]. This strategy has proven ineffective in cases of low endemicity and asymptomatic infections. Other authors have estimated that TBS only detects and controls 20–50% of cases [33,36]. Moreover, a community trial in Burkina Faso with 12 months of follow-up demonstrated that active detection strategy has no effect on the prevalence of *Plasmodium* spp. or the incidence of malaria [33,37] owing to the low sensitivity of the diagnosis. These and other factors have contributed to the need of evidence to make molecular diagnosis the standard, particularly in areas with low endemicity and transmission [33].

All of the above findings enable us to identify several problems that justify the need for this study:

(i) In the stages of malaria control, TBS effectiveness has only been demonstrated for the programmatic phase, ignoring its true value for the pre-elimination, elimination, and reintroduction prevention phases. In the case of Colombia, the CPG for malaria indicates that early diagnosis is a vital component of the control and elimination strategies as well as transmission reduction [17].
(ii) Although most experts believe that TBS should be used only in febrile patients, the central argument for this recommendation is not one of diagnostic accuracy or clinical–epidemiological rigor but of optimizing the scarce economic resources allocated to the epidemiological surveillance of this disease [1,11,17]. The impossibility in the short and medium term for Colombia to put molecular tests into operation in the places where patients arrive for consultation should also be considered.
(iii) In ideal epidemiological terms, a good diagnostic test for infectious diseases should cover the entire clinical spectrum of the disease, i.e., from its asymptomatic stages, rather than being limited to advanced stages where sensitivity and specificity are known to be high. For the epidemiological MAP surveillance, the diagnosis should not be limited to its clinical use in symptomatic patients, excluding the importance of a good diagnostic method for epidemiological surveillance of the infection and its transmission.
(iv) Colombian NIH recommends using TBS in pregnant women, who are usually asymptomatic, in their quarterly antenatal control, as well as screening of all people with an epidemiological link or exposure in an endemic area, demonstrating the need to be aware of the performance of TBS in this type of population.
(v) In Colombia, no diagnostic evaluation of all the accuracy parameters of TBS for symptomatic or asymptomatic pregnant women has been performed. Previous research shows high heterogeneity in diagnostic sensitivity in this group, ranging from 32.3% [27] to 63.7% [28]. TBS has been shown to have a high sensitivity in *P. falciparum* infections, but the parameters of accuracy in *P. vivax*-predominant settings are unknown [30].
(vi) The scant evidence on the diagnostic accuracy of TBS for MAP is focused on GM, with few approaches for PM and CM [31].
(vii) In Colombia, the effect of the following variables on the diagnostic evaluation parameters is unknown: causal species, anemic or febrile state, history of malaria in the last year, and history of GM, which are relevant to issuing recommendations on groups in which the TBS has a good or bad diagnostic performance.

Therefore, the aim of this research was to evaluate the accuracy of TBS versus quantitative PCR (qPCR) for the diagnosis of *P. falciparum* and *P. vivax* MAP in its GM, PM, and CM forms, and to compare the accuracy of TBS in different subgroups of pregnant women according to symptomatology (fever and anemia) and malaria history.

The importance of this type of research stems from the need for a valid test for the diagnosis in clinical and epidemiological surveillance of MAP, as its absence can lead to serious clinical and epidemiological situations, including the following:

(i) Without adequate and valid MAP calculations, physicians would either disregard negative results (usually false negatives [FNs]) or increase antimalarial drug prescriptions [38] owing to mistrust in their diagnostic standard. Other authors have reported that *Plasmodium* spp. was found in less than 1% of subjects receiving antimalarial treatment in low-endemic areas [39]. The clinical overdiagnosis of malaria in hospitals coexists with its underdiagnosis in the community, resulting in antimalarial drugs being administered to people who do not require them [40]. This would imply a misuse of resources allocated to treating malaria and an increased risk of antimalarial drug resistance [41].
(ii) Without community screening using valid and highly sensitive methods, surveillance would be limited to clinical disease, resulting in an inaccurate epidemiological and parasitological picture due to an underestimated prevalence and incidence as well as unreliable data for setting epidemiological surveillance, control, or elimination goals [42,43].
(iii) Without a proper diagnosis, the risk of cases of traveler’s malaria increases [44].

## 2. Materials and Methods

### 2.1. Study Subjects

We realized a diagnostic evaluation. The study subjects included 829 pregnant women, 579 placentas, 381 umbilical cord blood samples, and 221 peripheral blood samples from neonates collected in local hospitals in Colombia’s main malaria-endemic area (the area that reports the highest number of cases in the country), which is located in the northwestern part of the country, specifically in South Córdoba and northwest of Antioquia (Bajo Cauca and Urabá) [45]. The samples were collected between 2010 and 2019, using the same standardized procedures for the collection of information and the diagnosis of malaria. The sampling was non-probabilistic and the selection of the participants was carried out in the antenatal care service of each hospital.

The pregnant women were recruited at prenatal check-ups or during delivery and had to meet the following inclusion criteria: living in the municipality for at least 1 year, having TBS and qPCR test results for malaria, having no diagnosed diseases, having infections or complications during pregnancy, and signing an informed consent form. Only pregnant women who were receiving antimalarial treatment at the time of the study or 2 weeks prior to the study were excluded.

In most cases the samples obtained were not from the same subjects (pregnant woman, placenta, umbilical cord and neonate), therefore the results of GM, PM and CM are analyzed independently. This is due to multiple geographical, cultural, economic and health system-related (particularly functioning and coverage of antenatal care) barriers for this type of research in Colombia, which prevented the recruitment of pregnant women with their follow-up until the newborn.

### 2.2. Malaria Diagnosis

The TBS was used as an index test, and qPCR was used as a reference standard for diagnostic evaluation. For the TBS, a blood sample was obtained and a large globular drop was extracted from it. The drop was spread homogeneously on a slide sheet to cover an area of approximately 1 cm × 1 cm, dried at room temperature for 20 min, and then stained with modified Romanowsky’s stain. This stain is used in Colombia because of its stability and because it is an aqueous solution; therefore, it does not absorb humidity from the environment, making it suitable for hot and humid tropical climates. This test is considered positive when a minimum of 200 microscopic fields are observed under 100× magnification and at least one parasitic form is found. Based on this, the presence of *P. falciparum, P. vivax*, or mixed malaria is defined. The latter corresponds to the observation of parasitic forms of *P. vivax* and concomitant gametocytes of *P. falciparum* or regular asexual forms compatible with *P. falciparum* in a proportion of ≥40% of the 100 parasitic forms observed [18]. TBS accuracy depends on the quality of the smear on the slide and microscopist’s qualification, in this study TBS was carried out by microscopists of national malaria diagnostic network, they are health workers (nursing auxiliary or community health promoters) trained by the NIH in a course on TBS during three months, with certification and recertification by the Ministry of Health. Microscopists work exclusively to make TBS, and in this study they had more than 10 years of experience realizing this test. This guarantees an excellent diagnostic quality.

For qPCR, blood was drawn from the same sample used in the TBS, and disks of Whatman #3 filter paper were soaked for DNA extraction. PCR is the most widely used test for the molecular diagnosis of malaria, with a detection limit of <0.02 parasites/μL. The qPCR avoids post-amplification manipulation, allow for real-time monitoring of the amplification process, and quantify the number of microorganisms. It is based on fluorescent labels that demonstrate amplicon formation throughout the reaction, and the fluorescence intensity is related to the number of amplified products. For malaria, this test includes a highly conserved region of the *Plasmodium* 18S rRNA gene (4–8 copies of the 18S rRNA genes are expected in each *Plasmodium*) and a region in a gene common to all four *Plasmodium* species (*P. falciparum*, *P. vivax*, *P. malariae*, and *P. ovale*) that has conserved and polymorphic regions [33].

### 2.3. Collection of Information and Bias Control

TBS and qPCR tests on peripheral blood from the mother were used to diagnose GM. The same procedure (as in TBS and qPCR) was used to diagnose PM, but with placental blood. Likewise, peripheral blood samples from the neonate or the umbilical cord were used to diagnose CM. The following variables were extracted from the clinical history: febrile state, hemoglobin, and previous diagnosis of malaria (in the last year and in the current pregnancy).

To avoid partial verification bias, spectrum bias, and differential verification bias, the following criteria were applied: a group of patients representative of those receiving the diagnostic test in routine clinical practice was evaluated; the TBS and qPCR were applied to the same blood samples (samples taken at the same time); the diagnostic tests were processed independently and blindly (without knowing the results of the other test); and qPCR was used as the reference test in all cases.

### 2.4. Statistical Analysis

Absolute (#) and relative (%) frequencies were used to describe the groups. The frequency of GM, PM, and CM was determined based on the reference test (qPCR). The accuracy of the TBS for the diagnosis of GM was assessed using sensitivity parameters [true positive {TP}/(TP + FN)], specificity [true negative {TN}/(TN + false positive {FP})], positive predictive value [TP/(TP + FP)], negative predictive value [TN/(TN + FN)], the negative likelihood ratio [1 − Sensitivity/Specificity E i.e., FN/E], the Youden index [(S + E) − 1], and the validity index or proportion of patients correctly diagnosed [(TP + TN)/Total]. All of these parameters were calculated with 95% CI, and the positive likelihood ratio was not calculated given the absence of FP results with the TBS. These parameters were calculated for the entire group and the following subgroups: infected with *P*. *falciparum* or *P. vivax*, with or without fever, with or without anemia, with mild–moderate anemia, with or without malaria in the last year, and with or without a history of GM in the current pregnancy. The accuracy parameters were also calculated by exploring the association of the causal species with the symptoms and history of malaria. The same procedure was applied for PM. In the case of CM, it was not possible to estimate the accuracy parameters because all cases were detected using qPCR and no cases were detected using the TBS. Analyses were performed in EPIDAT (Dirección Xeral de Saúde Pública de la Consellería de Sanidade, Organización Panamericana de la Salud, Universidad CES de Colombia, Xunta de Galicia, España).

### 2.5. Ethical Aspects

The guidelines of the Declaration of Helsinki and Resolution 8430 of Colombia for research with pregnant women were followed. The study was classified as minimal risk and was approved by the Ethics Committee of the Sede de Investigación Universitaria, Universidad de Antioquia (Minutes # 21-101-961) and the Instituto de Investigaciones Médicas (Minutes # 008). All pregnant women signed the informed consent (for women of legal age) or assent (for women under 18 years of age) form, which was obtained in writing and signed by the pregnant woman and a witness (external to the research group). According to Colombian law, adolescents (over 10 years of age) may sign the informed consent form without the consent of their parents or guardians. All data were coded (anonymized) by someone outside the research team. To ensure the confidentiality of the data, the authors of this manuscript had access to a database that did not contain any information that could reveal the identity of the pregnant woman.

## 3. Results

Information was retrieved from the medical records of 1057 pregnant women with a history of malaria, with 32.8% (*n* = 347) having malaria in the last year and 25.1% (*n* = 265) in the current pregnancy. Among the 661 pregnant women, 19.1% (*n* = 126) reported having fever during the current malaria infection, and among the 792 patients with hemoglobin records, 31.3% (*n* = 248) had anemia (hemoglobin level < 11.0 g/dL) during gestation or at delivery.

There were 829 pregnant women who underwent qPCR, and 35.8% (*n* = 297) tested positive with the following results in terms of species: *P. vivax*, 63% (*n* = 187); *P. falciparum*, 34% (*n* = 102); and both species (mixed malaria), 3.0% (*n* = 8). The same 829 underwent TBS, 19.7% (*n* = 163) were positive for malaria (*P. vivax* in 79.8%, *P. falciparum* in 19.6%, and mixed in 0.6%) (Table 1).

Both tests were performed on 579 placental blood samples, and the results were as follows: qPCR revealed that 27.4% (*n* = 159) were positive, while the TBS revealed that 2.2% (*n* = 13) were positive. The species identified with qPCR were *P. falciparum*, 45.3% (*n* = 72); *P. vivax*, 40.9% (*n* = 65); and mixed, 13.8% (*n* = 22), while with the TBS, no mixed species were identified and the respective frequencies of *P. vivax* and *P. falciparum* were 69.2% (*n* = 9) and 30.8% (*n* = 4) (Table 1).

Two sources of blood samples were used to examine the newborn: the umbilical cord (at delivery) and peripheral blood (after delivery and up to the day of discharge, which is usually 1–2 days after birth). Only the qPCR data are presented because none of the children tested positive in the TBS. According to the neonatal peripheral blood samples, there were no mixed malaria, and there was a low frequency of *P. vivax* (*n* = 3) and *P. falciparum* (*n* = 2). According to the umbilical cord blood samples, 16.5% (*n* = 63) were positive, *P. falciparum* 63.5% (*n* = 40), *P. vivax* 30.2% (*n* = 19), and mixed malaria 6.3% (*n* = 4) (Table 1).

For GM, the TBS had a sensitivity of 55%, a specificity of 100%, a positive predictive value of 100%, and a negative predictive value of 79.9%. In the subgroups, the specificity and positive predictive values were both 100%. The lowest sensitivity values were found in those infected with *P. falciparum* (30%); pregnant women without fever (29%), without anemia (38.5%), or mild anemia (33%); and those without a history of malaria in the previous year (13%) or in the current pregnancy (20%). On the contrary, the best sensitivity results were found in those infected with *P. vivax* (68%), with a history of malaria (69%), and with febrile symptoms (96%). The lowest negative predictive values were recorded in pregnant women with anemia (66% in mild anemia and 57.5% in moderate anemia) and with a history of malaria (64% in those with GM in the current pregnancy and 76% in pregnant women who had malaria in the last year), while the highest values were found independent of diagnosis of species (88% in *P. falciparum* and 90% in *P. vivax*) or febrile state (89% in pregnant women without fever and 91% in febrile symptomatic women) (Table 2).

In the population evaluated for GM, the TBS had a negative likelihood ratio of 0.45, a Youden index value of 0.55, and a validity index (correctly diagnosed patients) of 83.8%. In the subgroup analyses, pregnant women with *P. falciparum* infection, no fever, and no history of malaria had the worst results in the negative likelihood ratio and Youden index. However, the best results in these two parameters were shown in pregnant women with fever, a history of malaria, and a *P. vivax* infection. The proportion of correctly diagnosed patients was higher in febrile symptomatic women and those infected with *P. vivax* (Table 3).

The diagnostic accuracy parameters for these cases could not be calculated because of the small number of mixed malaria results. It was also not possible to perform calculations according to the parasitemia reported in the TBS because parasite density was only obtained in 152 samples, with 35 pregnant women having less than 1000 asexual parasites/µL, 26 having 1000–2500 asexual parasites/µL, 30 having 2501–5000 asexual parasites/µL, and 61 having more than 5000 asexual parasites/µL. Both tests revealed that all of these samples were positive (i.e., in this subgroup of 152 pregnant women, parasitemia was not related to the diagnostic accuracy of the TBS).

The diagnostic errors in terms of species were as follows: one case of *P. falciparum* by TBS was a *P. vivax* infection on qPCR, one case of *P. falciparum* with TBS was a mixed malaria on qPCR, four cases of *P. vivax* on TBS were mixed malarias on qPCR, and one mixed malaria with TBS was a *P. falciparum* infection on qPCR.

*P. vivax* showed better results in terms of sensitivity, negative likelihood ratio, and in the Youden index. The negative predictive value for *P. falciparum* and *P. vivax* did not differ in any of the subgroups analyzed according to symptomatology and history of malaria. This parameter yielded the lowest results in pregnant women with anemia (80% in *P. falciparum* and 77% in *P. vivax*) and without malaria in the current pregnancy (50.0% in *P. falciparum* and 50.6% in *P. vivax*). The only subgroups with satisfactory diagnostic accuracy results were pregnant women with fever, regardless of the species, and those with a history of *P. vivax* malaria (in the last year or in the current pregnancy) (Table 4).

For PM, the sensitivity of the TBS was 8.2%; in subgroup analyses by causal species, symptomatology, and history of malaria, it ranged from 1.2% to 17.6%. The negative predictive value ranged from 49.3% to 93.9%, while the proportion of patients correctly diagnosed ranged from 52.0% to 94.0%, with the latter being explained by the absence of FPs in the TBS (Table 5). Specificity and positive predictive values were both 100% (no FPs). The negative likelihood ratio was greater than 0.80 and the Youden index value was less than 0.20 in all subgroups.

In 17 negative TBSs, mixed PM was diagnosed by qPCR. There was no analysis of diagnostic evaluation parameters in the subgroup of febrile pregnant women because only 33 cases were found, 29 of which were negative with both tests and four of which were negative with the TBS but positive with qPCR (the absence of FPs and TPs did not allow for the estimation of diagnostic evaluation parameters). Finally, the deficiencies of TBS in detecting PM cases in all subgroups analyzed according to species, symptomatology, and history of malaria were maintained in the estimation of diagnostic accuracy parameters for the interaction of these variables.

## 4. Discussion

In the study population, 19.1% of patients had febrile symptoms and 31.3% had anemia. These findings are consistent with the Health Situation Analysis of Colombia 2019, which reported a frequency of anemia of 28%, with anemia being 40% more frequent in the lowest wealth quintile [46], as well as with some reports on febrile syndromes in Colombia. These are the leading causes of emergency consultations in the country, with malaria being one of the main causes [47]. This represents the population in Colombia, where the TBS is commonly used.

### 4.1. Gestational Malaria

These findings apply to territories where *P. vivax* predominates, causing 63% of cases of GM (compared to 34% of *P. falciparum* and 3% of mixed malaria), which is novel in this field of research, where evaluations of diagnostic accuracy predominate in areas with a higher frequency of *P. falciparum* [28,29,30].

*P. vivax* predominated in GM as a reflection of the study region’s parasitological and epidemiological situations. However, in PM, both species had a similar proportion (45.3% for *P. falciparum* and 40.9% for *P. vivax*), whereas in CM, the proportion of *P. falciparum* was higher (62% vs. 32% for *P. vivax*), which would be supported by specific clinical, physiological, and pathological aspects of these species. Therefore, the similar proportion of *P. vivax* and *P. falciparum* in PM would contradict several assumptions about *P. vivax*’s lower capacity for clinical damage [20,21,22,48], while the higher proportion of CM cases by *P. falciparum* would reflect greater virulence, pathogenicity, and the ability to cross the placenta of this species [49].

The frequency of GM was 36%, PM 27%, and CM 2% in neonatal peripheral blood samples and 16.5% in umbilical cord samples, which are higher (except for CM) than those reported in a meta-analysis in the Colombian literature, with frequencies of 17%, 11%, and 16%, respectively [13]. Despite these differences, both studies report a high frequency of MAP cases with a high proportion of submicroscopic malaria (in this study, 45% of all cases of GM, 92% of PM, and 100% of CM), demonstrating the limitations of the TBS for MAP [13]. This finding is of concern for malaria care, control, and elimination programs because submicroscopic cases are not detected or treated, resulting in severe outcomes for the mother and child (anemia and severe malaria) while also perpetuating parasite transmission and disease endemicity [20,23,24,25,26].

For GM, TBS presented satisfactory diagnostic accuracy parameters for *P. vivax* infections (S = 68%; E = 100%, PPV = 100%, NPV = 90%; NLR = 0.3; and PPCD = 92%) in pregnant women with a history of malaria (S = 69%; E = 100%, PPV = 100%, NPV = 100%, NPV = 76%; NLR = 0.3; and PPCD = 84) and in febrile symptomatic women (S = 96%; E = 100%, PPV = 100%, NPV = 92%; NLR = 0.04; and PPCD = 97%). In this last case, this research makes no new contributions; rather, it confirms the excellent diagnostic capacity of the TBS in cases of advanced infection or parasitemia levels that cause obvious signs and symptoms, as indicated by WHO guidelines, the Colombian CPG, and some studies on MAP [1,11,17,29].

This study provides new evidence of the good diagnostic performance of the TBS for GM caused by *P. vivax* and in pregnant women with a history of malaria. Therefore, sensitivity and specificity demonstrate the test’s ability to detect positives and negatives without being affected by disease prevalence, implying that the results could be easily applied to settings with a different prevalence. These parameters are only affected by the disease spectrum, so patients with clearly manifested disease have better outcomes. In contrast, disease prevalence can influence the overall validity index or correctly classified subjects because accuracy increases as disease prevalence decreases [50].

The negative likelihood ratio (1 − S/E) is the ratio of the probability of a negative result in subjects with the disease to the probability of the same result in subjects without the disease (ideally it should be ≤0.1, but values of ≤0.3 are considered acceptable because this value is related to a change in clinical behavior). This is the most recommended measure for diagnostic accuracy because its sensitivity and specificity are independent of disease prevalence and can be used to calculate predictive values [50].

The lowest negative predictive values were found in pregnant women with anemia and a history of malaria, indicating that a negative TBS result in this type of pregnant woman is not very reliable for the clinician or treatment staff and that other diagnostic options should be explored when malaria is highly suspected and the TBS result is negative in order to prevent the infection from progressing. In clinical practice, a common line of reasoning is to know how good the test is at predicting disease or health. Therefore, predictive values are more commonly used, although they depend on disease prevalence [50].

In the other subgroups analyzed (*P. falciparum* cases and asymptomatic pregnant women or those with no history of malaria), TBS performed poorly. Furthermore, in these cases, other diagnostic options should be explored if progress is to be made in achieving the goals of pre-elimination, elimination, or prevention in the reintroduction of cases [32] and if the clinical consequences of submicroscopic and asymptomatic MAP (which some authors suggest should be referred to as chronic malaria) are to be avoided [23,24,25,26].

In addition, there are some difficulties in the diagnosis of mixed malaria using the TBS for GM, even in the case of diagnoses made by expert microscopists. In this study, six (86%) of seven cases of diagnostic mismatch by causal species were related to mixed malaria, which would generate errors in epidemiological and clinical profile.

### 4.2. Placenta Malaria

For PM, the sensitivity was 8%; in subgroup analyses according to species, symptoms, and history of malaria, it was 1–18%. The negative likelihood ratio (1 − S/E) was greater than 0.8 in all subgroups, indicating that with the TBS, the probability of a FN result (1 − S) is nearly equal to that of getting a TN result (E); this indicates that this test is not useful in any case or subgroup for PM [50]. This is consistent with the findings of other authors who have concluded that microscopy, placental histology, and other methods fail to identify the majority of *P. falciparum* infections detected by qPCR in peripheral blood and placenta. Undetected infections are associated with increased clinical risks, highlighting the need for improved MAP diagnosis and to avoid the consequences of hidden infections during pregnancy [51].

In pathophysiological terms, a factor for the control of MAP and its clinical consequences in the mother, fetus, and newborn revolves around PM, which is defined as the sequestration of *P. falciparum*-infected erythrocytes in the placenta or the presence of *Plasmodium* spp. or malarial pigment in this tissue, which can lead to reduced parasite circulation in the maternal peripheral blood and transmission to the fetus. PM is a difficult clinical form to diagnose (in fact, this diagnosis is restricted to the research field), with several studies demonstrating high variability in the sensitivity and specificity of qPCR and rapid diagnostic tests. Histopathology is the gold standard for PM diagnosis because it can detect both parasites and hemozoin. Histopathology can also detect immune cell infiltrates and classify placental tissue into acute active infection (presence of parasites with little or no pigment), chronic active infection (presence of parasites and abundant pigment), or past infection (only presence of pigment) based on the inflammatory process, which PCR cannot detect because it does not identify hemozoin [31].

However, this method is not without challenges because its results depend on the quality of the sample, and the formalin pigment can be confused with hemozoin, leading to diagnostic errors [5,52,53,54]. There are additional challenges, such as the fact that the histopathological damage that defines positivity for PM is unclear. Regardless, progress in the accurate diagnosis of PM before or during delivery is required not only for research purposes but also for programmatic purposes (for prenatal and malaria control programs) [5].

It is worth noting that a study conducted in the same region of this research compared the TBS, nested PCR, and histopathology for the diagnosis of MM, discovering a sensitivity of 33% and a specificity of 95% for the TBS, whereas in nested PCR, these parameters were 47% and 77%, respectively, when compared to histopathology [31].

### 4.3. Congenital Malaria

The TBS did not detect any cases, demonstrating the need for molecular diagnosis for this clinical form. Other studies have indicated that the diagnosis of CM is more complicated because it is an infection with low parasitic density in umbilical cord blood or peripheral blood of the newborn. Therefore, tests with better sensitivity, such as molecular techniques, are required [6]. In general, CM is a poorly investigated event, and some authors believe that it is a disease with low occurrence, so there are many gaps in the literature [7].

### 4.4. Limitations of This Study

Given the low number of cases, one of the study’s limitations is the lack of diagnostic accuracy analyses for mixed malaria. Furthermore, comprehensive analyses based on parasitemia levels were not conducted. Nevertheless, determination of parasitemia and classification are difficult and far from perfect; inaccurate diagnosis and classification have caused a high mortality rate, especially in children [51,55]. Although obtaining a blood sample via venipuncture could be considered a limitation, previous research has shown that there are no differences in parasite density or diagnostic yield between this type of sample and capillary blood [56], and the Colombian Ministry of Health recommendations validate both types of samples [18].

In terms of qPCR, one of its limitations is its inability to determine the viability of the microorganism because it only detects genetic material and does not identify individuals with the ability to transmit the agent and cause new infections. Therefore, two final considerations should be highlighted:

(i) in other models, such as the severe acute respiratory syndrome coronavirus 2 (SARS-CoV-2), the cycle threshold (Ct) of qPCR (semiquantitative measurement inversely related to the amount of genetic material in the sample) and its correlation with clinical data have been used to suggest reference ranges for Ct (<30, highly infectious; 30–34, moderately infectious; 35–37, indeterminate; and >37, non-infectious). Similar studies could be suggested for MAP [57], and
(ii) based on the outcomes previously mentioned for submicroscopic MAP cases, it can be assumed that the possibility of detecting asymptomatic carriage of malaria is low in the case of the molecular diagnosis of MAP. However, there is a clear need for further research and an increase in the health budget allocated to the molecular diagnosis of this disease.

### 4.5. Strengths of This Study

The following are the strengths of this study:

(i) It is Colombia’s first study with a large sample size for the mother–placenta–neonate trinomial.
(ii) It is one of the few studies in the world that presents the thorough diagnostic parameters for clinical and epidemiological programs in a comprehensive manner.
(iii) It assesses diagnostic ability in key subgroups according to symptoms, malaria history, and causal species and discusses the effect of the interaction of these factors on the accuracy of the TBS.
(iv) Although TBS was historically used in people with fever and PCR was developed to identify parasite components (DNA) in asymptomatic people or those with very low parasitemia, this study showed satisfactory results for cases of GM caused by *P. vivax* and in pregnant women with previous malaria, expanding the evidence in favor of the use of this test in various clinical and epidemiological situations (aspects for which there are no previous studies and demonstrate the novelty of this research). Moreover, there was no false positive in PM with TBS which is relevant to avoid subsequent resistance problems due to poor prescription of antimalarials. However, in GM, classification errors occurred in 0.8% mainly in cases of mixed GM; despite being a very low number, it shows the importance of carrying out more diagnostic accuracy studies for mixed malaria.
(v) Evidence was gathered to support the importance of active surveillance of MAP in Colombia, which requires more effort compared to passive detection because health workers must search for patients in the community. This type of search is crucial for the pre-elimination and elimination phases of malaria because it allows for the detection of undiagnosed symptomatic cases with passive surveillance, as well as asymptomatic patients using focus studies.

## 5. Conclusions

The TBS performed well in this study for the diagnosis of GM in pregnant women with *P. vivax* infection, previous malaria and fever (or symptoms). It also demonstrated that the TBS performed poorly to detect PM and CM because of its low case-detection capacity. It is necessary to conduct MAP surveillance, follow-up, and control with molecular diagnosis in order to improve the detection of asymptomatic GM and *P. falciparum* in pregnant women with no history of malaria, optimize the timely treatment of PM and CM, avoid the clinical consequences of MAP.

## Figures and Tables

**Table 1:** Description of the study population according to the source (mother, placenta, or neonate) *Plasmodium*.

			*n*	%
**Pregnant woman**	**qPCR** *n* = 829	Total	297	35.8
		*P. falciparum*	102	12.3
		*P. vivax*	187	22.5
		Mixed	8	1.0
	**TBS** *n* = 829	Total	163	19.7
		*P. falciparum*	32	3.9
		*P. vivax*	130	15.7
		Mixed	1	0.1
**Placenta**	**qPCR** *n* = 579	Total	159	27.4
		*P. falciparum*	72	12.4
		*P. vivax*	65	11.2
		Mixed	22	3.8
	**TBS** *n* = 579	Total	13	2.2
		*P. falciparum*	4	0.7
		*P. vivax*	9	1.5
**Neonate**	**qPCR peripheral blood** *n* = 221	Total	5	2.3
		*P. falciparum*	2	0.9
		*P. vivax*	3	1.4
	**qPCR** **Umbilical cord** *n* = 381	Total	63	16.5
		*P. falciparum*	40	10.5
		*P. vivax*	19	5.0
		Mixed	4	1.0

**Table 2:** Frequency, sensitivity, specificity, and predictive values of the TBS compared to qPCR for the diagnosis of gestational malaria in the total population and in subgroups according to species, symptomatology, and history of malaria.

	Frequency	S	E	PPV	NPV
**Total** (positive vs. negative for malaria)					
% (95% CI)	35.8 (32.5–39.1)	54.9 (49.0–60.7)	100 (99.9–100)	100 (99.7–100)	79.9 (79.7–83.0)
N	297/829	163/297	532/532	163/163	532/666
**By species** (positive for species vs. negative for malaria)					
*P. falciparum*					
% (95% CI)	16.0 (13.0–18.9)	29.7 (20.3–39.1)	100 (99.9–100)	100 (98.3–100)	88.2 (85.6–90.9)
n	101/633	30/101	532/532	30/30	532/603
*P. vivax*					
% (95% CI)	25.9 (22.6–29.2)	67.7 (60.8–74.7)	100 (99.9–100)	100 (99.6–100)	89.9 (87.4–92.4)
n	186/718	126/186	532/532	126/126	532/592
**By Symptom** (positive for each symptom vs. negative for the symptom)					
No fever					
% (95% CI)	14.9 (10.9–18.9)	28.6 (14.9–42.2)	100 (99.8–100)	100 (96.4–100)	88.9 (85.3–92.5)
n	49/329	14/49	280/280	14/14	280/315
With fever					
% (95% CI)	68.9 (59.5–78.3)	95.8 (90.4–100)	100 (98.4–100)	100 (99.3–100)	91.4 (80.7–100)
n	71/103	68/71	32/32	68/68	32/35
No anemia					
% (95% CI)	26.8 (22.1–31.5)	38.5 (28.3–48.8)	100 (99.8–100)	100 (98.6–100)	81.6 (77.2–86.0)
n	96/358	37/96	262/262	37/37	262/321
With anemia					
% (95% CI)	49.1 (41.3–56.9)	41.0 (29.8–52.2)	100 (99.4–100)	100 (98.5–100)	63.7 (55.2–72.2)
n	83/169	34/83	86/86	34/34	86/135
With mild anemia					
% (95% CI)	43.2 (33.6–52.9)	33.3 (19.0–47.7)	100 (99.2–100)	100 (96.9–100)	66.3 (56.3–76.3)
n	48/111	16/48	63/63	16/16	63/95
With moderate anemia					
% (95% CI)	60.3 (46.9–73.8)	51.4 (33.4–69.4)	100 (97.8–100)	100 (97.2–100)	57.5 (40.9–74.1)
n	35/58	18/35	23/23	18/18	23/40
**Presence of a history of malaria**					
With a history of malaria in the last year					
% (95% CI)	50.5 (44.5–56.6)	69.0 (61.1–77.0)	100 (99.6–100)	100 (99.5–100)	76.0 (69.5–82.4)
N	142/281	98/142	139/139	98/98	139/183
No history of malaria in the past year					
% (95% CI)	19.8 (16.1–23.4)	12.6 (5.4–19.8)	100 (99.9–100)	100 (95.8–100)	82.3 (78.7–85.9)
N	95/481	12/95	386/386	12/12	386/469
With a history of malaria in this pregnancy					
% (95% CI)	64.0 (57.1–70.9)	68.8 (60.3–77.2)	100 (99.3–100)	100 (99.4–100)	64.3 (55.0–73.6)
n	128/200	88/128	72/72	88/88	72/112
No history of malaria in this pregnancy					
% (95% CI)	19.4 (16.0–22.7)	20.2 (12.2–28.2)	100 (99.9–100)	100 (97.7–100)	83.9 (80.7–87.1)
n	109/562	22/109	453/453	22/22	453/540

F: frequency of GM, S: sensitivity, E: specificity, PPV: positive predictive value, NPV, negative predictive value.

**Table 3:** Likelihood ratios, validity indices, and Youden index values of the TBS compared to qPCR for the diagnosis of gestational malaria in the total population and in subgroups according to species, symptomatology, and history of malaria.

	NLR	Youden	PPCD
**Total**	0.45 (0.40–0.51)	0.55 (0.49–0.61)	83.8 (81.3–86.4)
**By species**			
*P. falciparum*	0.70 (0.62–0.80)	0.30 (0.21–0.39)	88.8 (86.3–91.3)
*P. vivax*	0.32 (0.26–0.40)	0.68 (0.61–0.74)	91.6 (89.5–93.7)
**By symptom**			
No fever	0.71 (0.60–0.85)	0.29 (0.16–0.41)	89.4 (85.9–92.9)
With fever	0.04 (0.01–0.13)	0.96 (0.91–1.00)	97.1 (93.4–100)
No anemia	0.61 (0.52–0.72)	0.39 (0.29–0.48)	83.5 (79.5–87.5)
With anemia	0.59 (0.49–0.71)	0.41 (0.30–0.52)	71.0 (63.9–78.1)
With mild anemia	0.67 (0.55–0.81)	0.33 (0.20–0.47)	71.2 (62.3–80.1)
With moderate anemia	0.49 (0.35–0.68)	0.51 (0.35–0.68)	70.7 (58.1–83.3)
**By history of malaria**			
With malaria during the last year	0.31 (0.24–0.40)	0.69 (0.61–0.77)	84.3 (79.9–88.8)
No malaria during last year	0.87 (0.81–0.94)	0.13 (0.06–0.19)	82.7 (79.3–86.2)
With malaria during this pregnancy	0.31 (0.24–0.40)	0.69 (0.61–0.77)	80.0 (74.2–85.8)
No malaria during this pregnancy	0.80 (0.73–0.88)	0.20 (0.13–0.28)	84.5 (81.4–87.6)

NLR: negative likelihood ratio, PPCD: proportion of patients correctly diagnosed (validity index).

**Table 4:** Diagnostic accuracy parameters of the TBS against qPCR for *Plasmodium falciparum* and *Plasmodium vivax* in pregnant women according to symptoms and history of malaria.

	S	NPV	NLR	Younden
No fever				
*P. falciparum*	18.2 (0–36.6) *n* = 4/22	94.0 (91.1–96.8) *n* = 280/298	0.82 (0.67–1.0)	0.18 (0.0–0.34)
*P. vivax*	40.0 (18.8–61.2) *n* = 10/25	94.9 (92.2–97.7) *n* = 280/305	0.60 (0.44–0.83)	0.40 (0.21–0.59)
With fever				
*P. falciparum*	100 (75.0–100) *n* = 2/2	100 (98.0–100) *n* = 32/32	--	1.0 (1.0–1.0)
*P. vivax*	95.6 (90.1–100) *n* = 66/69	91.4 (80.7–100) *n* = 32/35	0.04 (0.01–0.13)	0.96 (0.91–1.0)
No anemia				
*P. falciparum*	20.9 (7.6–34.2) *n* = 9/43	88.5(84.7–92.3) *n* = 262/296	0.79 (0.68–0.92)	0.21 (0.09–0.33)
*P. vivax*	50.0 (34.8–65.2) *n* = 24/48	91.6(88.2–95.0) *n* = 262/286	0.50 (0.38–0.66)	0.50 (0.36–0.64)
With anemia				
*P. falciparum*	4.3 (0–14.9) *n* = 1/23	79.6(71.6–87.7) *n* = 86/108	0.96 (0.88–1.0)	0.04 (0–0.13)
*P. vivax*	55.2 (41.5–68.8) *n* = 32/58	76.8 (68.5–85.0) *n* = 86/112	0.45 (0.34–0.60)	0.55 (0.42–0.68)
With malaria in the last year				
*P. falciparum*	25.0 (8.4–41.6) *n* = 8/32	85.3 (79.5–91.0) *n* = 139/163	0.75 (0.61–0.92)	0.25 (0.10–0.40)
*P. vivax*	81.9 (74.1–89.7) *n* = 86/105	88.0 (82.6–93.4) 139/158	0.18 (0.12–0.27)	0.82 (0.75–0.89)
No malaria during last year				
*P. falciparum*	6.8 (0–15.4) *n* = 3/44	90.4 (87.5–93.3) *n* = 386/427	0.93 (0.86–1.0)	0.07 (0–0.14)
*P. vivax*	16.7 (5.1–28.3) *n* = 8/48	90.6 (87.7–93.5) 386/426	0.83 (0.73–0.95)	0.17 (0.06–0.27)
With malaria in this pregnancy				
*P. falciparum*	18.5 (2.0–35.0) *n* = 5/27	76.6 (67.5–85.7) *n* = 72/94	0.81 (0.68–0.98)	0.19 (0.04–0.33)
*P. vivax*	82.3 (74.1–90.4) *n* = 79/96	80.9 (72.2–89.6) *n* = 72/89	0.18 (0.12–0.27)	0.82 (0.75–0.90)
No malaria in this pregnancy				
*P. falciparum*	12.2 (2.0–22.4) *n* = 6/49	50.0 (38.8–61.1) *n* = 43/86	0.88 (0.79–0.97)	0.12 (0.03–0.21)
*P. vivax*	26.3 (14.0–38.6) *n* = 15/57	50.6 (39.4–61.8) *n* = 43/85	0.74 (0.63–0.86)	0.26 (0.15–0.38)

S: sensitivity; NPV: negative predictive value, NLR: negative likelihood ratio. Specificity and positive predictive value were 100%. The proportion of patients correctly diagnosed (validity index) was greater than 80% in all subgroups (explained by the absence of false positives).

**Table 5:** Diagnostic evaluation parameters of the TBS compared to qPCR for the diagnosis of placental malaria in the total population and in subgroups according to species, symptomatology, and history of malaria.

	Frequency	S	NPV	PPCD
**Total**				
% (95% CI)	27.5 (27.3–27.6)	8.2 (7.8–8.5)	74.2 (74.1–74.3)	74.8 (74.7–74.9)
n	159/579	13/159	420/566	(13 + 420)/579
**By species**				
*P. falciparum*				
% (95% CI)	14.6 (14.5–14.7)	5.6 (4.8–6.3)	86.1 (85.9–86.2)	86.2 (86.1–86.3)
n	72/492	4/72	420/492	(4 + 420)/492
*P. vivax*				
% (95% CI)	13.4 (13.3–13.5)	6.1 (5.3–7.0)	87.3 (87.2–87.4)	87.4 (87.3–87.5)
n	65/485	4/65	420/481	(4 + 420)/485
**By symptomatology**				
No anemia				
% (95% CI)	24.0 (24.8–25.2)	9.7 (9.0–10.5)	76.9 (76.7–77.1)	77.4 (77.2–77.6)
n	72/288	7/72	216/281	(7 + 216)/288
With anemia				
% (95%CI)	52.2 (51.7–52.7)	10.2 (9.3–11.1)	50.5 (49.9–51.0)	53.1 (52.6–53.6)
n	59/113	6/59	54/107	(6 + 54)/113
With mild anemia				
% (95% CI)	53.4 (52.7–54.2)	10.3 (8.9–11.6)	49.3 (48.5–50.1)	52.0 (51.3–52.8)
n	39/73	4/39	34/69	(4 + 34)/73
With moderate anemia				
% (95% CI)	50.0 (48.7–51.3)	10.0 (7.4–12.6)	52.6 (51.2–54.0)	55.0 (53.7–56.3)
n	20/40	2/20	20/38	(2 + 20)/40
No fever				
% (95% CI)	6.5 (6.3–6.8)	7.7 (3.8–11.6)	93.9 (93.7–94.2)	94.0 (93.7–94.2)
n	13/199	1/13	186/198	(1 + 186)/199
**Presence of a history of malaria**				
No malaria last year				
% (95% CI)	18.9 (18.7–19.0)	1.3 (0.6–1.9)	81.3 (81.2–81.4)	81.3 (81.2–81.5)
n	78/413	1/78	335/412	(1 + 335)/413
With malaria last year				
% (95% CI)	47.3 (46.9–47.7)	17.1 (16.4–17.9)	57.3 (56.9–57.8)	60.8 (60.4–61.2)
n	70/148	12/70	78/136	(12 + 78)/148
No malaria in this pregnancy				
% (95% CI)	18.6 (18.4–18.7)	1.2 (0.6–1.9)	81.6 (81.5–81.7)	81.7 (81.5–81.8)
n	80/431	1/80	351/430	(1 + 351)/431
With malaria in this pregnancy				
% (95% CI)	52.3 (51.9–52.7)	17.6 (16.9–18.4)	52.5 (52.1–53.0)	56.9 (56.5–57.4)
n	68/130	12/68	62/118	(12 + 62)/130

F: frequency of gestational malaria, S: sensitivity, NPV: negative predictive value, PPCD: proportion of patients correctly diagnosed (validity index).

## Data Availability

All relevant data is cited in the manuscript.

## References

[1] World Health Organization (2022). World Malaria Report 2022. https://www.who.int/publications/i/item/9789240040496.

[2] González M. (2014). Malaria and pregnancy: Complications, prevention and treatment. Prog. Obstet. Ginecol..

[3] Malaria Policy Advisory Group World Health Organization (2017). Meeting Report of the Evidence Review Group on Malaria in Pregnancy, Geneva, Switzerland, 9–11 July 2017.

[4] Carmona-Fonseca J. (2020). The epidemiology of malaria in Colombia: A heretical view. Soc. Med..

[5] Liu Y., Griffin J.B., Muehlenbachs A., Rogerson S.J., Bailis A.J., Sharma R., Sullivan D.J., Tshefu A.K., Landis S.H., Kabongo J.M. (2016). Diagnosis of placental malaria in poorly fixed and processed placental tissue. Malar. J..

[6] Omer S.A., Adam I., Noureldien A., Elhaj H., Guerrero-Latorre L., Silgado A., Sulleiro E., Molina I. (2020). Congenital malaria in newborns delivered to mothers with malaria-infected placenta in Blue Nile State, Sudan. J. Trop. Pediatr..

[7] Gülaşı S., Özdener N. (2016). Congenital malaria: Importance of diagnosis and treatment in pregnancy. Turk. J. Pediatr..

[8] Carmona-Fonseca J., Maestre A. (2009). Incidencia de Las Malarias Gestacional, Congénita y Placentaria en Urabá (Antioquia, Colombia), 2005–2007. Rev. Colomb. Obstet. Ginecol..

[9] Centers for Disease Control and Prevention (2022). Intermittent Preventive Treatment of Malaria for Pregnant Women (IPTp). https://www.cdc.gov/malaria/malaria_worldwide/reduction/iptp.html#:~:text=Malaria%20infection%20during%20pregnancy%20can,a%20risk%20factor%20for%20death.

[10] Carmona-Fonseca J., Cardona-Arias J.A. (2022). Placental malaria caused by *Plasmodium vivax* or *P. falciparum* in Colombia: Histopathology and mediators in placental processes. PLoS ONE.

[11] World Health Organization (2015). Guidelines for Treatment of Malaria.

[12] Carmona-Fonseca J., Cardona-Arias J.A. (2021). Overview of epidemiology of malaria associated with pregnancy in northwestern Colombia, 1985–2020. J. Commun. Dis..

[13] Cardona J., Carmona J. (2021). Meta-analysis of the prevalence of malaria associated with pregnancy in Colombia 2000-2020. PLoS ONE.

[14] Cardona J., Carmona J. (2022). Frequency of gestational malaria and maternal–neonatal outcomes, in Northwestern Colombia 2009–2020. Sci. Rep..

[15] Cardona J., Carmona J. (2022). Frequency of placental malaria and its associated factors in northwestern Colombia, pooled analysis 2009–2020. PLoS ONE.

[16] Cardona J., Carmona J. (2022). Congenital malaria: Frequency and epidemiology in Colombia, 2009–2020. PLoS ONE.

[17] Ministerio de Salud y Protección Social de Colombia (2022). Guía de Práctica Clínica Diagnóstico y Tratamiento de la Malaria.

[18] Instituto Nacional de Salud de Colombia (2015). Manual Para el Diagnóstico de Malaria no Complicada en Puestos de Diagnóstico y Tratamiento.

[19] Cortés L.J., Guerra A.P. (2020). Concordance analysis of three diagnostic tests for malaria in the symptomatic population of Colombian endemic municipalities. Biomedica.

[20] Gavina K., Gnidehou S., Arango E., Hamel-Martineau C., Mitran C., Agudelo O., Lopez C., Karidio A., Banman S., Carmona-Fonseca J. (2018). Clinical outcomes of submicroscopic infections and correlates of protection of VAR2CSA antibodies in a longitudinal study of pregnant women in Colombia. Infect. Immun..

[21] López C., Carmona J. (2020). Malaria placentaria submicroscópica: Histopatología y expresión de mediadores de procesos fisiológicos. Rev. Peru. Med. Exp. Salud Pública.

[22] Agudelo O., Arango E., Carmona J. (2017). Submicroscopic and asymptomatic congenital infection by *Plasmodium vivax* or *P. falciparum* in Colombia: 37 cases with placental histopathology and cytokine profile in maternal and placental blood. J. Trop. Med..

[23] Buchwald A.G., Sixpence A., Chimenya M., Damson M., Sorkin J.D., Wilson M.L., Seydel K., Hochman S., Mathanga D.P., Taylor T.E. (2019). Clinical implications of asymptomatic *Plasmodium falciparum* infections in Malawi. Clin. Infect. Dis..

[24] Nguyen T.N., von Seidlein L., Nguyen T.V., Truong P.N., Hung S.D., Pham H.T., Nguyen T.U., Le T.D., Dao V.H., Mukaka M. (2018). The persistence and oscillations of submicroscopic *Plasmodium falciparum* and *Plasmodium vivax* infections over time in Vietnam: An open cohort study. Lancet Infect. Dis..

[25] Chen I., Clarke S.E., Gosling R., Hamainza B., Killeen G., Magill A., O’Meara W., Price R.N., Riley E.M. (2016). “Asymptomatic” Malaria: A chronic and debilitating infection that should be treated. PLoS Med..

[26] Lin J.T., Saunders D.L., Meshnick S.R. (2014). The role of submicroscopic parasitemia in malaria transmission: What is the evidence?. Trends Parasitol..

[27] Ahmed R., Levy E.I., Maratina S.S., de Jong J.J., Asih P.B., Rozi I.E., Hawley W., Syafruddin D., ter Kuile F. (2015). Performance of four HRP-2/pLDH combination rapid diagnostic tests and field microscopy as screening tests for malaria in pregnancy in Indonesia: A cross-sectional study. Malar. J..

[28] Umbers A.J., Unger H.W., Rosanas-Urgell A., Wangnapi R.A., Kattenberg J.H., Jally S., Silim S., Lufele E., Karl S., Ome-Kaius M. (2015). Accuracy of an HRP-2/panLDH rapid diagnostic test to detect peripheral and placental *Plasmodium falciparum* infection in Papua New Guinean women with anaemia or suspected malaria. Malar. J..

[29] Kashif A.H., Adam G.K., Mohmmed A.A., Elzaki S.E., AbdelHalim A.M., Adam I. (2013). Reliability of rapid diagnostic test for diagnosing peripheral and placental malaria in an area of unstable malaria transmission in Eastern Sudan. Diagn. Pathol..

[30] Rogerson S.J., Mkundika P., Kanjala M.K. (2003). Diagnosis of *Plasmodium falciparum* malaria at delivery: Comparison of blood film preparation methods and of blood films with histology. J. Clin. Microbiol..

[31] Campos I.M., Uribe M.L., Cuesta C., Franco-Gallego A., Carmona-Fonseca J., Maestre A. (2011). Diagnosis of gestational, congenital, and placental malaria in Colombia: Comparison of the efficacy of microscopy, nested polymerase chain reaction, and histopathology. Am. J. Trop. Med. Hyg..

[32] Oyegoke O.O., Maharaj L., Akoniyon O.P., Kwoji I., Roux A.T., Adewumi T.S., Maharaj R., Oyebola B.T., Adeleke M.A., Okpeku M. (2022). Malaria diagnostic methods with the elimination goal in view. Parasitol. Res..

[33] Zheng Z., Cheng Z. (2017). Advances in molecular diagnosis of malaria. Adv. Clin. Chem..

[34] Cao J., Sturrock H.J., Cotter C., Zhou S., Zhou H., Liu Y., Tang L., Gosling R.D., Feachem R.G., Gao Q. (2014). Communicating and monitoring surveillance and response activities for malaria elimination: China’s “1-3-7” strategy. PLoS Med..

[35] Sturrock H.J., Hsiang M.S., Cohen J.M., Smith D.L., Greenhouse B., Bousema T., Gosling R.D. (2013). Targeting asymptomatic malaria infections: Active surveillance in control and elimination. PLoS Med..

[36] Cotter C., Sturrock H.J., Hsiang M.S., Liu J., Phillips A.A., Hwang J., Gueye C.S., Fullman N., Gosling R.D., Feachem R.G. (2013). The changing epidemiology of malaria elimination: New strategies for new challenges. Lancet.

[37] Tiono A.B., Ouédraogo A., Ogutu B., Diarra A., Coulibaly S., Gansané A., Sirima S.B., O’Neil G., Mukhopadhyay A., Hamed K. (2013). A controlled, parallel, cluster-randomized trial of community-wide screening and treatment of asymptomatic carriers of *Plasmodium falciparum* in Burkina Faso. Malar. J..

[38] Sansom C. (2009). Overprescribing of antimalarials. Lancet Infect. Dis..

[39] Reyburn H., Mbakilwa H., Mwangi R., Mwerinde O., Olomi R., Drakeley C., Whitty C.J. (2007). Rapid diagnostic tests compared with malaria microscopy for guiding outpatient treatment of febrile illness in Tanzania: Randomised trial. BMJ.

[40] Ansah E.K., Narh-Bana S., Epokor M., Akanpigbiam S., Quartey A.A., Gyapong J., Whitty C.J. (2010). Rapid testing for malaria in settings where microscopy is available and peripheral clinics where only presumptive treatment is available: A randomised controlled trial in Ghana. BMJ.

[41] Lubell Y., Reyburn H., Mbakilwa H., Mwangi R., Chonya S., Whitty C.J., Mills A. (2008). The impact of response to the results of diagnostic tests for malaria: Cost-benefit analysis. BMJ.

[42] Cibulskis R.E., Aregawi M., Williams R., Otten M., Dye C. (2011). Worldwide incidence of malaria in 2009: Estimates, time trends, and a critique of methods. PLoS Med..

[43] Harris I., Sharrock W.W., Bain L.M., Gray K.A., Bobogare A., Boaz L., Lilley K., Krause D., Vallely A., Johnson M.L. (2010). A large proportion of asymptomatic *Plasmodium* infections with low and sub-microscopic parasite densities in the low transmission setting of Temotu Province, Solomon Islands: Challenges for malaria diagnostics in an elimination setting. Malar. J..

[44] malERA Consultative Group on Diagnoses and Diagnostics (2011). A research agenda for malaria eradication: Diagnoses and diagnostics. PLoS Med..

[45] Carmona J. (2017). La Región “Urabá Antioqueño-Cuencas altas de los ríos Sinú y San Jorge-Bajo Cauca Antioqueño”: “guarida” del paludismo colombiano Revista de la Universidad Industrial de Santander. Salud.

[46] Ministerio de Salud de Colombia (2019). Análisis de Situación de Salud (ASIS) Colombia, 2019. https://www.minsalud.gov.co/sites/rid/Lists/BibliotecaDigital/RIDE/VS/ED/PSP/asis-2019-colombia.pdf.

[47] Cortés J., Romero L., Aguirre C., Pinzón L., Cuervo S. (2017). Enfoque clínico del síndrome febril agudo en Colombia. Infectio.

[48] Dayananda K.K., Achur R.N., Gowda D.C. (2018). Epidemiology, drug resistance, and pathophysiology of *Plasmodium vivax* malaria. J. Vector Borne Dis..

[49] Moxon C.A., Gibbins M.P., McGuinness D., Milner D.A., Marti M. (2020). New insights into malaria pathogenesis. Annu. Rev. Pathol..

[50] Eusebi P. (2013). Diagnostic accuracy measures. Cerebrovasc. Dis..

[51] Mayor A., Moro L., Aguilar R., Bardají A., Cisteró P., Serra-Casas E., Sigaúque B., Alonso P.L., Ordi J., Menéndez C. (2012). How hidden can malaria be in pregnant women? Diagnosis by microscopy, placental histology, polymerase chain reaction and detection of histidine-rich protein 2 in plasma. Clin Infect. Dis..

[52] Fried M., Muehlenbachs A., Duffy P.E. (2012). Diagnosing malaria in pregnancy: An update. Expert Rev. Anti-Infect. Ther..

[53] Takem E.N., D’Alessandro U. (2013). Malaria in pregnancy. Mediterr. J. Hematol. Infect. Dis..

[54] Romagosa C., Menendez C., Ismail M.R., Quintó L., Ferrer B., Alonso P.L., Ordi J. (2004). Polarisation microscopy increases the sensitivity of hemozoin and *Plasmodium* detection in the histological assessment of placental malaria. Acta Trop..

[55] Rehman A., Abbas N., Saba T., Mehmood Z., Mahmood T., Ahmed K.T. (2018). Microscopic malaria parasitemia diagnosis and grading on benchmark datasets. Microsc. Res. Tech..

[56] Solari L., Soto A., Mendoza D., Llanos A. (2002). Comparación de las densidades parasitarias en gota gruesa de sangre venosa versus digitopunción en el diagnóstico de Malaria Vivax. Rev. Méd. Herediana.

[57] Serrano A., Ruiz A., Segura A., Olmo V., Micó R., Barquilla A., Morán A. (2021). Aplicación del valor umbral del número de ciclos (Ct) de PCR en la COVID-19. Semergen.

